# Mental Time Travel in Post-Traumatic Stress Disorder: Current Gaps and Future Directions

**DOI:** 10.3389/fpsyg.2021.624707

**Published:** 2021-03-09

**Authors:** Nadia Rahman, Adam D. Brown

**Affiliations:** ^1^Department of Psychology, The New School for Social Research, New York, NY, United States; ^2^Department of Psychiatry, New York University School of Medicine, New York, NY, United States

**Keywords:** post-traumatic stress disorder (PTSD), autobiographical memory, prospection, future thinking, cognition, mental time travel, flashbacks, flashforwards

## Introduction

Exposure to potentially traumatic events is a common experience throughout the world. Although many people typically do not experience mental health issues following such events, a significant minority of individuals will develop a psychiatric disorder. Post-traumatic stress disorder (PTSD) is the most frequent negative mental health outcome of traumatic events (e.g., Hoge et al., 2004) and is associated with considerable functional impairment (e.g., Silverstein et al., 2018). Although recent decades have been marked by important advances in PTSD treatments, a subset of patients still does not respond to first-line pharmacotherapy or extant evidence-based psychotherapies (Lancaster et al., 2016), underscoring the need for new treatment development. Elucidation of the neurocognitive processes underlying the pathogenesis and maintenance of PTSD may represent a critical step toward developing more optimal treatments.

Among the neurocognitive processes that have been shown to be altered in individuals with PTSD are autobiographical memories (AMs), a class of self-referential, long-term memories comprised of both episodic and semantic details. AMs may be retrieved deliberately (voluntarily) or unexpectedly (involuntarily). AMs are critical for wellbeing and functioning, as they are implicated in numerous clinically-relevant processes, including problem-solving; the creation of self-concept; and the formation and maintenance of social-bonds (Bluck et al., 2005)— all processes that are dysregulated in PTSD (e.g., Lord et al., 2020). Given the integral role of AM in PTSD, it is unsurprising that numerous studies have examined AM alterations related to this disorder. One such alteration that has received considerable attention is known as the overgeneral memory (OGM), a phenomenon in which individuals demonstrate difficulty recalling discreet, discontinuous autobiographical events. That is, an AM marked by reduced specificity, which may take place repeatedly or over the course of several days. OGM has been shown to be a predictor of posttraumatic symptoms (Moore and Zoellner, 2007) and an effective target for therapy. Indeed, AM specificity training has been shown to achieve PTSD symptom reduction in half the number of sessions required using standard cognitive processing therapy treatments (e.g., Maxwell, 2016). In addition to OGM, other AM alterations have been implicated in PTSD, including changes in the emotional intensity associated with the recollection of a particular type of memory (e.g., Rubin, 2010; Niziurski et al., 2017); variations in the availability and coherence of different memory types (e.g., Rubin et al., 2008); increases in intrusive, emotionally-laden memories and prospections (e.g., Berntsen and Rubin, 2015); and greater centrality of traumatic experiences to self-identity (e.g., Berntsen and Rubin, 2006).

An important function of the episodic memory system is the construction of prospective memories, also known as episodic future-thinking (EFT). Through a phenomenon known as mental time travel (MTT; Tulving, 1985), we are not only able to experience ourselves in the past, but we are also able to experience ourselves in the future. Building upon MTT theory, Schacter and Addis (2007) put forth the *Constructive Episodic Simulation Hypothesis*, which posits that the cognitive processes of remembering the past and thinking about the future rely on similar underlying mechanisms, and both draw upon information stored in episodic memory. Episodic memory and EFT operate by binding the context of an experience to the experience itself for the construction and reconstruction of scenes (Schacter and Addis, 2007). Importantly, this theory suggests that EFT relies on the flexible recombination of information drawn from past experiences to construct novel future scenarios. While this scenario construction can be adaptive for processes such as planning and goal-attainment, clinically-relevant biases in AM may contribute to the onset and maintenance of various disorders, including PTSD (Hallford et al., 2018).

MTT is thought to serve several critical functions, including decision-making (Aupperle et al., 2011), problem-solving (Suddendorf and Corballis, 1997), and goal-setting and implementation (Breeden et al., 2016). However, further research is needed to elucidate the relationship between PTSD and alterations in MTT. While the examination of the AM component of MTT is ubiquitous in PTSD research, there is a relative dearth of information on the future thinking component of this process (however, see, e.g., Blix and Brennen, 2011; Zlomuzica et al., 2017). In line with the *Constructive Episodic Simulation Hypothesis*, these studies show that putative AM alterations associated with PTSD are also found in the content and characteristics of future imaginings among such individuals. The relevance of EFT and the construction of a future self in relation to psychopathology converges with a growing body of research demonstrating that prospection related to the self is associated with a wide range of cognitive, affective, and behavioral processes. For instance, we tend to evaluate ourselves in the present by comparing ourselves to the future self we hope to achieve (Bak, 2015). Relatedly, neurocognitive findings consistently link PTSD with negative self-appraisals (Engelbrecht and Jobson, 2020).

The goal of the current article is to highlight the importance of MTT in PTSD. Although changes in MTT may reflect a mechanism underlying several mental health disorders, both the study and the treatment of PTSD have long focused on potential changes in the characteristics and content in AM. Given this history and the long-standing debates related to the role of memory in this disorder, as well as the targeted use of AM in treatments (e.g., Brewin, 2011), this commentary will be limited to PTSD. However, alterations in AM are by no means constrained to PTSD, and certain MTT changes may reflect transdiagnostic processes (e.g., Kellogg et al., 2020). With this opinion piece, we will (1) briefly review and consider the extant literature on memory and future-thinking in PTSD; (2) elucidate gaps in PTSD MTT research; (3) discuss future directions for PTSD MTT research. It is our hope that this piece will shed light on an understudied cognitive process and underscore areas where additional research may broaden our understanding of PTSD pathogenesis and eventually inform the development of future PTSD treatments.

## Alterations in Memory and Future Thinking in PTSD

### Voluntariness, Intrusiveness, and Content

Considerable AM research in PTSD has focused on memories that arise spontaneously, namely, involuntary AMs, intrusive AMs, and flashbacks. Although these terms are often used interchangeably in research studies, recent attempts to create a taxonomy have been employed to study potentially distinct phenomena associated with these processes (Kvavilashvili, 2014). For instance, Kvavilashvili suggests that involuntary AMs arise spontaneously without attempt at recall; may occur in response to internal and external triggers; and can be positive, negative, or neutral in valence (e.g., Berntsen and Hall, 2004). Intrusive AMs, by contrast, may be spontaneous memories of predominantly negative events that repeatedly interrupt consciousness, often emerging against one's will, and may interrupt one's activity at the time. Compared to intrusive AMs, involuntary AMs may be more random in nature and will often go unnoticed. Lastly, flashbacks might represent a type of spontaneous and intrusive memory specifically related to a traumatic event. Flashbacks have been characterized as vivid, emotionally-laden, and lacking temporal perspective, or possessing a “here and now” like quality (e.g., Zlomuzica et al., 2017). Future research into these potential distinctions may not only help to better characterize the phenomenology of PTSD but also to refine treatments aimed at reducing distress related to such AM processes.

Although there remains debate in the field about the extent to which involuntary memories and flashbacks may or may not reflect poorly integrated memories, there is now substantial evidence in line with cognitive models of PTSD indicating that stressful memories, such as those experienced by individuals with PTSD, are more, not less, accessible. Moreover, it is likely due to the increased availability of these highly stressful memories that individuals with PTSD are more likely to recall trauma-related memories. Interestingly, research has revealed that this is true for involuntary and voluntary AMs (Berntsen, 2010). For example, several studies have shown that while individuals with PTSD may think more about their trauma-related involuntary memories and experience greater subjective reactions to these memories, they do so for voluntary trauma-related AM's as well (e.g., Berntsen, 2001; Rubin, 2010). Despite the growing body of work elucidating the basic cognitive processes underlying involuntary AMs in PTSD, considerable debate and calls for additional research into the distinct qualities of flashbacks in PTSD continue (Brewin, 2015).

PTSD studies have also sought to investigate the differential effects of trauma on the encoding and recollection of voluntary and involuntary AM's. Disagreement in the field regarding this topic is centered around two assumptions about PTSD symptomatology: (1) traumatic events are frequently reexperienced via involuntary flashbacks, and (2) voluntary access to traumatic memory is impaired such that it is partially to completely inaccessible. These PTSD characteristics have been included in the DSM-V (American Psychiatric Association, 2013) are proposed in theoretical models of PTSD (e.g., Ehlers and Clark, 2000). Howeverindings pertaining to accessibility of traumatic AMs have indicated that both involuntary *and* voluntary memory have been shown to be enhanced by stressful events (e.g., Hall and Berntsen, 2008). Moreover, this enhancement has been associated with increased levels of PTSD symptoms (e.g., Rubin et al., 2008). Given the persistence and scope of this debate, future research would benefit from comparing voluntary and involuntary memories in individuals with reliable PTSD diagnoses (Berntsen and Rubin, 2014), using mixed-methodologies to examine differences between memory types. Of particular interest would be the further employment of task-based methodologies that allow for the measurement of intrusive memories in real-time, such as the trauma-film paradigm (e.g., Holmes and Bourne, 2008) or the AM diary recording method (e.g., Schönfeld and Ehlers, 2017).

The extent to which involuntary AM's are specific or not may depend on whether that AM is related to trauma. For example, Schönfeld and Ehlers (2017) found that compared to individuals without PTSD, traumatic AM's appeared more specific, as they were experienced more vividly and were associated with a subjective sense of “nowness.” However, the group with PTSD perceived their traumatic memories as less intentional, indicating that while traumatic and nontraumatic memories may be similarly involuntary post-trauma, individuals with PTSD may overestimate the frequency of trauma-related involuntary memories because they are noticed more often than non-trauma related involuntary memories (Kvavilashvili, 2014). By contrast, nontraumatic memories in the group with PTSD were associated with reduced continuity between past self and present self; and were less specific (Schönfeld and Ehlers, 2017). The potential differences in specificity for involuntary AMs related and unrelated to trauma are intriguing and may help to explain the Janus-like phenomenon often attributed to PTSD, where on the one hand, individuals with PTSD recall highly vivid, trauma-related AMs and on the other appear to have difficulty recalling other memories, perhaps unrelated to the trauma.

Future episodic intrusions have received considerably less attention than flashbacks. In their investigation of mental imagery as a predictor of future suicidal behavior, Holmes et al. (2007) found that depressed, formerly-suicidal patients unanimously reported ruminating on the imagery of future suicide attempts. The authors termed these future-related intrusions, *flashforwards*, because of their similarity to flashbacks. Both trauma-related flashforwards and flashbacks are emotionally-laden, contain rich detail, and evoke a phenomenological sense of “nowness.” Survivors of Chernobyl's nuclear disaster similarly reported flashforwards, projecting themselves into futures in which they develop health problems caused by their prior radiation exposure (Loganovsky and Zdanevich, 2013). Closely related to this phenomenon, Berntsen and Rubin (2015) examined the construct of *pretraumatic stress* in combat veterans before, during, and after deployment. Pretraumatic stress involves future-directed thoughts about what might be experienced over the course of combat. Interestingly, pretraumatic stress reactions were reliable predictors of PTSD both during and after combat-related trauma exposure. Additionally, flashforwards, heightened arousal, and associated avoidance behavior were experienced at the same level of post-traumatic stress reactions to traumatic events experienced before and during deployment, indicating that pretraumatic stress and posttraumatic stress may represent two subjectively distinct manifestations of the same underlying phenomenon. Together, these studies point to potential therapeutic targets in imagery features of flashbacks and flashforwards. One such intervention to consider is imagery rescripting, a method that involves focusing on imagery to modify the meaning of a traumatic event (for review, see Hackmann, 2011). Additionally, although studies have indicated that future autobiographical imaginings tend to be overgeneralized in response to non-trauma related cues, to our knowledge, no studies have examined whether flashforwards for non-trauma-related or trauma-related in PTSD are also less specific.

### Coherence

As previously noted, it has been consistently demonstrated that individuals with PTSD exhibit overgeneralized AM for voluntarily-recalled, nontraumatic memories (Moore and Zoellner, 2007; Ono et al., 2015). However, findings regarding alterations in voluntarily-recalled, traumatic memories in individuals with PTSD are mixed. Certain studies show increased fragmentation and disorganization in voluntarily recalled traumatic memories in individuals with PTSD (e.g., Halligan et al., 2003), while other studies show no significant group differences in the qualities of voluntarily-recalled traumatic memories (e.g., Jelinek et al., 2009).

The extant literature on memory and PTSD reveals conflicting findings regarding whether or not PTSD involves disorganization and fragmentation of memory. The *basic mechanisms view* proposes that there is heightened availability of trauma or stressful AMs compared to non-stressful or traumatic AMs, regardless of whether this memory is retrieved voluntarily or involuntarily (Rubin et al., 2008). Although other researchers have argued in favor of models that support fragmented memories (e.g., Monroe and Mineka, 2008) and lack of control narratives, variance in education level and narrative skill make it challenging to determine lack of coherence found in several studies (Gray and Lombardo, 2001; McNally, 2003). Ongoing research is needed to fully appreciate how AMs are shaped by trauma, as following a trauma, certain parts of an event may be enhanced while others become harder to recall. For example, one study found that negative emotion enhances memory for items central to the event but diminishes coherence for non-central, contextual items around the traumatic event (e.g., Bisby et al., 2018).

The *special mechanism view* (e.g., van der Kolk and Fisler, 1995) hypothesizes that there is disrupted encoding, integration, and voluntary retrieval of memories around a traumatic event. For example, a recent study found that the presence of fragmentation and disorganization across a trauma narrative is positively associated with emotional intensity surrounding that trauma. That is to say, highly emotional moments in a traumatic event are more likely to be associated with a disruption in cognitive processing that in turn creates disorganization and fragmentation (Brewin, 2015). Further research is needed to clarify which types of memories— trauma vs. general AMs, involuntary vs. voluntary memories—if any are characterized by disorganization and fragmentation.

There is very little PTSD research on disorganization and fragmentation of prospections, and the few studies that do exist show mixed results. Two studies specifically have examined temporal distribution in both AM and future thinking. Zlomuzica et al., 2017 tested MTT and temporal distribution using a novel virtual reality paradigm that probed not only episodic content but spatiotemporal context in MTT. When compared to their healthy counterparts, PTSD participants performed worse overall in the MTT test, and specifically on items that probed questions of *when*? and *what*? Significantly, PTSD participants were less able to employ information from episodic memory to solve current or future problems, and when emotional arousal was probed, they had higher levels of negative arousal and lower levels of positive arousal. By contrast, Blix and Brennen (2011) examined the relationship between trauma exposure and temporal distribution in AMs and prospection and found no group differences between trauma-exposed participants and healthy controls without a PTSD diagnosis. Additionally, it will be helpful for future research to examine other dimensions of future self-coherence. For instance, a growing body of literature has found that greater future self-continuity, the extent to which you believe your current self overlaps with your future self, is associated with reduced impulsivity and positive health outcomes (Hershfield, 2011). To our knowledge, no studies have looked at whether this aspect of self may be altered in PTSD and how it might be related to the specificity of characteristics of MTT.

### Specificity and Vividness

When recalling the past, individuals with PTSD reliably demonstrate OGM bias. Several theories attempt to explain the overgeneralization of AM seen in individuals with PTSD (for reviews, see Moore and Zoellner, 2007; Ono et al., 2015). This effect has been shown in both traumatic and nontraumatic memories (Schönfeld and Ehlers, 2017), using a range of methodologies both inside and outside the laboratory (e.g., Brown et al., 2013; Schönfeld and Ehlers, 2017). Notably, OGM prompted by positive cues was shown to be a risk factor for post-traumatic stress after a traumatic experience (Bryant et al., 2008), indicating a possible negativity bias in individuals with PTSD.

Recent PTSD research shows a similar pattern of overgeneralization in EFT in a range of post-traumatic populations with heterogeneous forms of trauma exposure using different methodologies (e.g., Brown et al., 2013; Zlomuzica et al., 2017). One study found that this effect was only present in response to positive prompts about the future (Kleim et al., 2014). Further research needs to be done to clarify whether overgeneralization of EFT in individuals with PTSD is specific to the valence of the cue eliciting the response and whether the overgeneralization of EFT remains during spontaneous EFT.

It was recently shown that individuals with PTSD tend to generate greater external detail (e.g., non-episodic details including semantic information, repetitions, and metacognitive statements) than they do internal detail (e.g., episodic information about the central event across a range of modalities including perceptual, emotional, spatial, and temporal) during memory retrieval and EFT (Brown et al., 2014). Although preliminary, these results show that in addition to overgeneralization of the spatial and temporal features of an event, internal detail also lacks specificity during MTT. New research is showing that cognitive processes related to depression can be altered using specificity training for the future (Hallford et al., 2020), and it will be important to determine whether such findings will generalize to PTSD.

### Changes in Self-Concept and Event Centrality

Cognitive frameworks of memory posit that one of its functions may be to inform and update our self-conception (Conway and Pleydell-Pearce, 2000). Conway and colleagues' seminal *Self-Memory-System Model* proposes that our goals and self-conceptions are constrained by AMs that contradict our current self-views. Given the central role of memory in PTSD, it is unsurprising then that traumatic memories are associated with negative beliefs about one's self in that past, present, and future (Beck, 1976). Cognitive frameworks relating memory to self are supported by experimental evidence. For example, PTSD has been associated with subjective feelings of increased societal alienation and permanent changes in the self across diverse trauma-exposed populations (Brewin, 2011); PTSD patients endorsed significantly more trauma-related memories when prompted to recall a self-defining memory than their trauma-exposed individuals without PTSD (Sutherland and Bryant, 2005); and trauma centrality has consistently been associated with greater PTSD symptomatology (e.g., Brown et al., 2010). Jobson et al. (2014) found that PTSD participants recalling AMs across a range of individualist and collectivist cultures showed similar reductions in expressions of autonomy and self-determination. In addition, longitudinal studies have demonstrated negative changes in self-perception significantly predict PTSD status follow-up (Kleim et al., 2007) as well as diminished response to exposure therapy (Ehlers et al., 1998).

In contrast to past and present self-conceptions, less is known about how our future self-concept and future goals are affected by PTSD. The *Self-Memory-System Model* predicts that future goals should be congruent with current symptomology. There is considerably less experimental evidence supporting the relationship between PTSD and changes in future self-conception. One study found that PTSD patients experienced disorder-congruent changes in cognitive tasks related to MTT and that trauma related-memories were significantly predicted by future goals related to trauma (Krans et al., 2017). In an experimental manipulation, Brown and colleagues demonstrated that self-efficacy, a facet of our self-identity, can be promoted by boosting AMs involving self-efficacious behavior in a sample of veterans with PTSD. Importantly, veterans in the enhanced self-efficacy condition exhibited enhanced social problem-solving and imagined the future with more goal-oriented statements than those in the control condition (Brown et al., 2016). Taken together, these findings suggest that negative self-conceptions may be modifiable and may represent a potential therapeutic target for future interventions.

Another facet of self-identity that has become an important correlate in PTSD research relates to how one views a traumatic event within the narrative of their life story. There is now considerable research showing that individuals who view their trauma as more central to their past and future and who view the event(s) as a turning point in their life will exhibit greater PTSD symptom severity. It may be that considering an event as more central increases the accessibility of those trauma memories, leading to PTSD-related symptoms. In contrast, it might be that events that are more profound and disruptive may also be seen as more central. Regardless of the directionality, studies that employ the Centrality of Event Scale (CES; Berntsen and Rubin, 2006) have found positive associations between the CES and PTSD symptoms (e.g., Brown et al., 2010; Rubin et al., 2014). One study attempted to reduce PTSD symptoms by experimentally manipulating event centrality (Vermeulen et al., 2019). Although symptoms of PTSD did not decrease, centrality did. Notably, other research using a modified version of Acceptance and Commitment Therapy (ACT) found that reductions in trauma centrality contributed to a decrease of PTSD, suggesting that trauma centrality may be modifiable, which in turn, may contribute to recovery from a traumatic stressor (Boals and Murrell, 2016). Such strategies are similar to those used in treatments such as Narrative Exposure Therapy (Robjant and Fazel, 2010), but additional research would help to understand the specific putative therapeutic mechanisms.

Although less is known about the relationship between PTSD and the centrality for future anticipated events, there are reasons to believe that similar trauma-focused biases about the past would apply to the future. First, Robinaugh and McNally (2011) did a principal component analysis on the CES and found that the future-oriented items on the CES were significantly associated with PTSD severity. Notably, this future-related factor was more strongly associated with PTSD symptom severity than the other two factors identified by the principal component analysis: factor 1, the extent to which the trauma is viewed as a turning point in one's life, and factor 2, the extent to which the trauma is integrated with one's other memories and its centrality to identity. Taken together, these findings indicate that the perception of trauma as central to one's future may be more damaging than the perception of trauma as central to one's current self. Second, studies examining temporal self-appraisals in PTSD show that while trauma-exposed individuals without PTSD hold a relatively optimistic view of the future, this is less often the case among those with PTSD (Brown et al., 2011; Parnes et al., 2020). As mentioned, re-scripting future anticipated events, perhaps related to pretraumatic stress, may help to interrupt some of the trauma-related expectations held by individuals with PTSD.

## Discussion and Future Directions

Over the last few decades, research on memory specificity has illuminated important mechanisms underlying PTSD, which in turn have guided novel treatments for the disorder (Maxwell, 2016). Further research using a wider range of neurocognitive methodologies is necessary to elucidate the key mechanisms underlying the relationship between MTT and PTSD. Such research is critical, as MTT may account for the social and functional impairments related to the disorder. In particular, future studies should investigate the role of the future-self in PTSD. Given the close relations between MTT and self-identity, therapeutic approaches that target MTT and self-narrative, such as imagery rescripting (Brockman and Calvert, 2017), metacognition (Davis et al., 2016), self-efficacy enhancement (Cusack et al., 2019), and specificity training for memory and prospection (Erten and Brown, 2018) may represent interventions and should be further tested in clinical PTSD populations.

## Author Contributions

All authors listed have made a substantial, direct and intellectual contribution to the work, and approved it for publication.

## Conflict of Interest

The authors declare that the research was conducted in the absence of any commercial or financial relationships that could be construed as a potential conflict of interest.
